# Uncovering hidden structures: previously undescribed pseudopodia and ectoplasmic structures in planktonic foraminifera

**DOI:** 10.1093/plankt/fbad031

**Published:** 2023-07-05

**Authors:** Mattia Greco, Adele Westgård, Freya E Sykes, Mohamed M Ezat, Julie Meilland

**Affiliations:** Institute of Marine Sciences (ICM), CSIC, Passeig MARítim de la Barceloneta, 37-49, Barcelona 08003, Spain; Cage – Centre for Arctic Gas Hydrate, Environment and Climate, Department of Geosciences, UIT, The Arctic University of Norway, Dramsveien 201, Tromso 9010, Norway; Cage – Centre for Arctic Gas Hydrate, Environment and Climate, Department of Geosciences, UIT, The Arctic University of Norway, Dramsveien 201, Tromso 9010, Norway; Cage – Centre for Arctic Gas Hydrate, Environment and Climate, Department of Geosciences, UIT, The Arctic University of Norway, Dramsveien 201, Tromso 9010, Norway; Marum – Center for Marine Environmental Sciences, University of Bremen, Leoberner Str. 8, Bremen 28359, Germany

**Keywords:** planktonic foraminifera, ectoplasmic roots, trophic strategy, laboratory observations

## Abstract

The trophic strategies of cold-water planktonic foraminifera are not well understood due to the challenge of culturing them in polar conditions. Here, we identify previously unknown ectoplasmic and cytoplasmic projections in three species of planktonic foraminifera thriving in polar and subpolar marine environments: *Globigerina bulloides*, *Neogloboquadrina incompta* and *Neogloboquadrina pachyderma*. These structures were observed during routine monitoring of cultured specimens sampled from the Norwegian coast, Greenland Sea and Baffin Bay. Two types of projections were discovered, including permanent and non-permanent structures such as ectoplasmic roots, twigs and twig-like projections, similar to those observed in benthic taxa *Cibicides* and *Cibicidoides*. Additionally, a previously undescribed filopodia-like projection was observed in *N. pachyderma*. We discuss the function, the ecological significance and the potential impact on pelagic processes of the presence of these structures in foraminifera species that occupy diverse niches in the water column. Our findings suggest that these structures may play an important role in the trophic strategies of cold-water planktonic foraminifera, and further research and observations are necessary to fully comprehend their significance in the carbon cycle.

## INTRODUCTION

Eukaryotic microbes exhibit a remarkable array of structural adaptations that enable them to carry out a diverse range of complex behaviors including defense and attack mechanisms, infection, modes of locomotion, feeding, reproduction, buoyancy control and more (Keeling and del Campo, 2017). By leveraging their structural diversity, these microorganisms can execute remarkable repertoire strategies essential for their survival and success in various ecological niches.

Foraminifera (Rhizaria) are microbial eukaryotes thriving in aquatic environments and soil that use cytoplasmic projections named reticulopodia (or rhizopodia) as the primary means of locomotion (Travis *et al.*, 2002; Holzmann *et al.*, 2021). These structures, once extended in the surrounding environment, can anastomose, creating complex networks of pseudopodia characterized by a constant bidirectional cytoplasmic flow (Hedley *et al.*, 1967; Bowser and Travis, 2002). The movement of these web-like formations is powered by cytoplasmic microtubules that also enable intracellular transport, generating the tracks that regulate the bidirectional transit of the organelles (Travis *et al.*, 2002).

In the pelagic environment, the abrupt shed of reticulopodia allows planktonic species to reduce the fluid drag and move downward in the water column (Furbish and Arnold, 1997). However, the exact mechanism of how planktonic foraminifera achieve and regulate their buoyancy throughout their life cycle is poorly understood.

Next to reticulopodia, other cytoplasmic protrusions have been described in foraminifera, including frothy pseudopodia involved in the calcification process and reported in both benthic and planktonic species (Schiebel and Hemleben, 2017; Tyszka *et al.*, 2019). In addition, structures named filopodia and axopodia have been identified in planktonic foraminifera (Adshead, 1966; Schiebel and Hemleben, 2017), but notably, the adoption of these terms varied across investigations and has been recently revisited (Zlatogursky, 2021).

The transport of food particles is one of the major functions of reticulopodia; however, for benthic taxa that live in the sediment, they also represent a vital substrate adhesion apparatus (Goldstein, 1999). A recent study has shown that, in some deep-sea benthic species, reticulopodia can build permanent formations, called ectoplasmic structures, that serve as an anchor in highly dynamic ecosystems and that, when conditions change, the foraminifera leaves behind (Wollenburg *et al.*, 2021). Importantly, since they have only been observed in benthic species of the family Cibicididae, interpretations of ectoplasmic structures’ ecological and evolutionary significance have been limited to their specific habitat (i.e. the deep-sea marine sediment) (Wollenburg *et al*., 2021).

To date, no evidence of the occurrence of such structures has been reported in any planktonic species.

Here, we describe for the first time overlooked ectoplasmic structures and cytoplasmic projections in the planktonic foraminifera species *Globigerina bulloides*, *Neogloboquadrina incompta* and *Neogloboquadrina pachyderma* sampled off the coast of Norway, in the Greenland Sea and in the Baffin Bay. We serendipitously observed these structures in cultured specimens during routine monitoring of their rhizopodial activity in the autumn of 2018 (Greco *et al.*, 2020) and in the summer of 2021 and 2022 (Ezat *et al.*, 2022a, 2022b). Life-history observations on these species are particularly scarce given the high complexity of keeping cold-adapted foraminifera alive in culture (Manno *et al.*, 2012). Additionally, cultivating Non-Spinose foraminifera of the *Neogloboquadrina* genus is challenging (Von Langen *et al*., 2005), primarily due to their small size. These species behave differently from Spinose foraminifera, such as *G. bulloides*, as they tend to stick to the walls of the flask rather than floating in the culture media (e.g. Davis *et al*., 2017; Fehrenbacher *et al*., 2018). The wide distribution of these species in the water column (Rebotim *et al.*, 2017; Greco *et al.*, 2019) suggests a need to reconsider the function of foraminiferal ectoplasmic structures in a pelagic context. The pelagic ecosystem comprises a multitude of micro-habitats (Boero *et al.*, 2019), such as sea ice and marine snow, where planktonic foraminifera are known to thrive (Dieckmann *et al.*, 1991; Fehrenbacher *et al.*, 2018; Greco *et al.*, 2021). By describing ectoplasmic structures and novel projections, we can enhance our understanding of planktonic foraminiferal trophic strategies and explore how this behavior could affect foraminifera-related carbon fluxes, as well as buoyancy control. Thus, this paper aims to document the existence of different cytoplasmic projections and ectoplasmic structures in planktonic species, discuss their ecological significance and explore their potential impact on pelagic processes, including intra and interspecific interactions and carbon export.

## METHODS

### Sampling and experimental settings

Sample collection and experimental setup are described in detail by Greco *et al*., 2020 and Westgård *et al*. (in prep). Briefly, specimens of *N. incompta*, *N. pachyderma and G. bulloides* were collected during three cruises on the RV Helmer Hanssen in autumn 2018 and summer 2021 and 2022, respectively, conducted off Tromsø (Greco *et al*., 2020, Ezat *et al*., 2022b), in the Greenland Sea (Ezat *et al.*, 2022a) and at the end of summer 2022, in the Baffin Bay onboard the RV Maria S. Merian (Table I). Specimens were sampled from the production zone, between 0 and 50 or 100 m, using a WP2 plankton net (64-μm mesh size, HydroBios) towed vertically or a multi net equipped with a 100-μm mesh size (Multi Plankton Sampler, HydroBios type Midi). The retrieved specimens were picked on board and incubated in jars (70 or 150 mL) containing seawater collected at the site of collection and filtered through a 0.22-μm nitrate cellulose filter. Temperature and salinity were kept such that they would mimic the environmental conditions. The collected foraminifera were then transferred to different temperature and salinity treatments for specific scientific projects (Greco *et al.*, 2020; Meilland *et al.*, 2022; Westegård *et al*. in prep) and kept in a cold room directly onboard and/or in one of the facilities of UiT—The Arctic University of Norway in Tromsø or in incubators where experiments and microscope observations were performed. Cytoplasm-bearing specimens were cultured individually in 75 mL Falcon flasks or in 10 mL wells (culture Figures) and under constant temperature (2, 4.5, 6, 7, 9 or 9.5°C), salinity (31, 32.1, 34.8, 35, 36.7) and pH (7.8, 8, and 8.1) conditions. All specimens were exposed to their *in situ* light cycles (e.g. Manno *et al*., 2012; Greco *et al*., 2020). They were fed daily with autoclaved marine microalgae *Nannochloropsis* food mix (30–50 μL *Nannochloropsis* concentrate: 200 mL filtered seawater), which had undergone autoclave steps to prevent bacteria and algal proliferation, or with living diatoms (*Pseudonitzschia turgidula*). This approach aimed to simulate a diet that closely mimicked their natural environment. The water of all *N. pachyderma* and *G. bulloides* specimens sampled and cultured in 2022 was partly or fully replaced once a week to ensure stable carbonate chemistry.

**Table I:** Sampling area of origin and culturing conditions of the foraminifera specimens included in the study

Species	ID	Year	Origin	t (°C)	pH	Salinity	Day	Structure	Figure
*N. incompta*	B-h2	2018	Norwegian Sea	6	–	31	13–18	Root	Fig. 1
*N. pachyderma*	89	2021	Greenland Sea	5	8	34.8	18	Root	Fig. 2e
*N. pachyderma*	295	2021	Greenland Sea	5	8	34.8	11	Root	Fig. 2f
*N. pachyderma*	254	2021	Greenland Sea	5	8	34.8	4	Filopodia-like	Fig. 2d
*N. pachyderma*	4	2021	Greenland Sea	6	7.8	35	40	Twig-like	Fig. 2b
*N. pachyderma*	15	2021	Greenland Sea	2	8.1	35	21	Twig-like	Fig. 2a
*N. incompta*	B-h1	2018	Norwegian Sea	6	-	31	12	Root fragment	Fig. 2c
*N. pachyderma*	9	2021	Greenland Sea	6	8.1	36.7	26	Filopodia-like	Fig. 3
*N. pachyderma*	11	2021	Greenland Sea	6	8.1	32.1	16	Twig-like	Supplementary Fig. 1c–d
*N. pachyderma*	257	2022	Baffin Bay	5	8	34.8	11	Twig-like	Fig. 4
*G. bulloides*	Gc100_18	2022	Norwegian Sea	10	8.1	35	18	Twigs	Fig. 5
*N. pachyderma*	C250	2021	Greenland Sea	6	8.1	35	17	Root and filopodia-like	Supplementary Fig. 1b
*N. pachyderma*	12	2021	Greenland Sea	9	8.1	35	4	Twig-like	Supplementary Fig. 1a

All specimens were checked at least bi-weekly under the inverted microscope (AxioVert 0.1, Zeiss), and information relative to their cytoplasm color, rhizopodial extent and activities were recorded.

All measurements were performed in ImageJ v1.8.0 (Schneider *et al.*, 2012).

Since no standardized methodology exists for characterizing the diversity, composition and potential role of reticulopodia in planktonic foraminifera, we decided to use the terminology from research on benthic species and other microbial groups to describe our observations.

## RESULTS

### Ectoplasmic roots in *N. incompta*

**Fig. 1:**
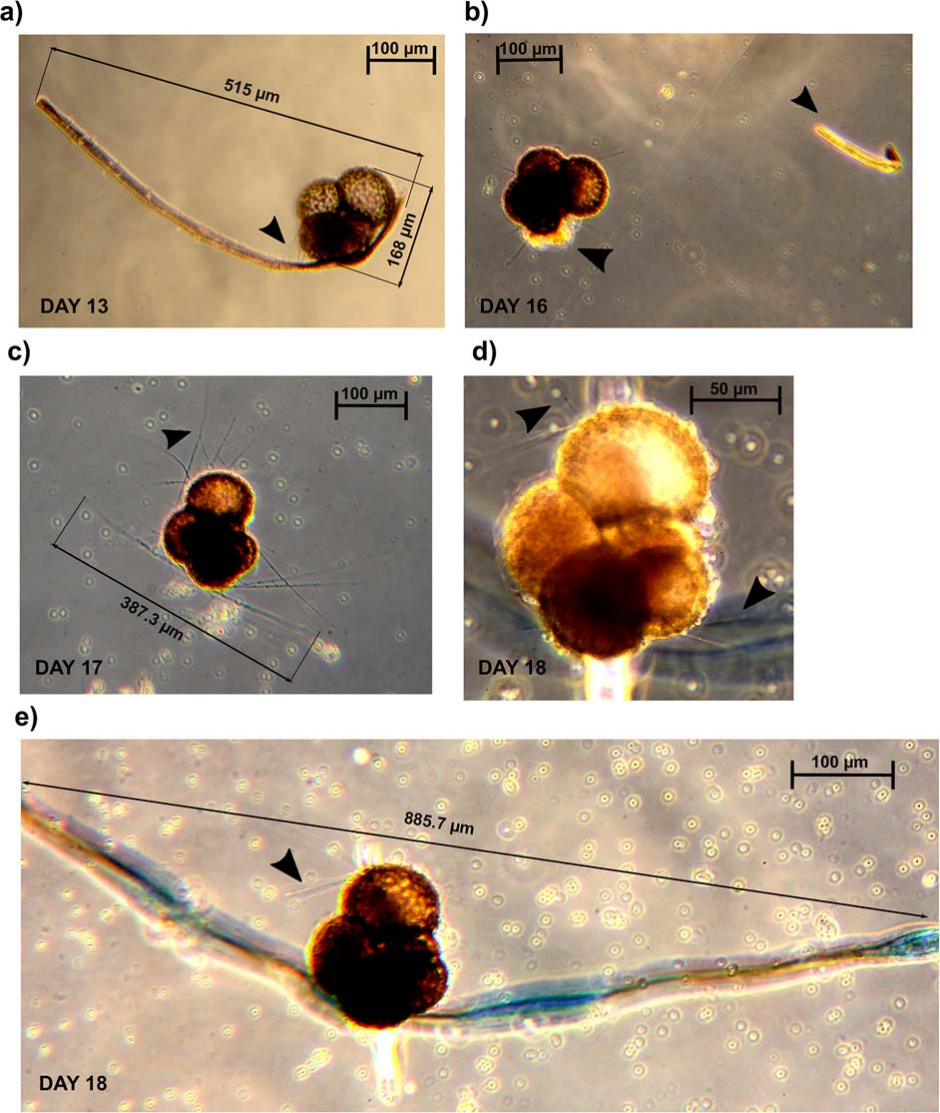
Serial observations of a cultured specimen of *Neogloboquadrina incompta* collected in the Norwegian Sea presenting and forming ectoplasmic roots. Panels (**a**) to (**e**) depict different stages of ectoplasmic root formation observed over the course of 6 days in culture. Black arrows in panels (a), (**c**), (**d**) and (e) indicate the rhizopodial activity. The black arrows in panel (b) indicate the root fragment and the location of the fracture on the test. The contrast in the pictures has been artificially enhanced to visualize the reticulopodia and the ectoplasmic roots. Scale bars: 100 μm.

**Fig. 2:**
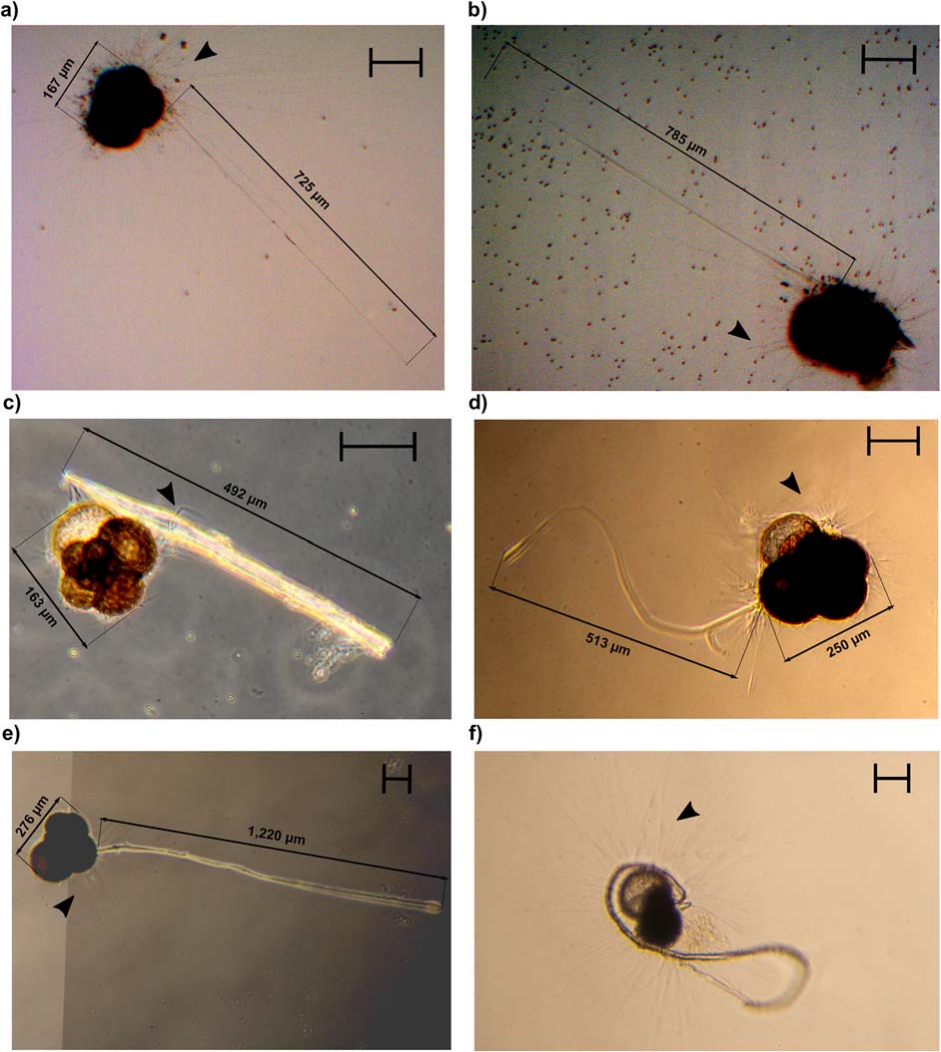
Permanent and Non-Permanent structures observed in specimens of the Genus *Neogloboquadrina*. Panels (**a**) and (**b**) depict two *Neogloboquadrina pachyderma* specimens displaying extended twig-like structures. The *Neogloboquadrina incompta* specimen in panel (**c**) is attached to an ectoplasmic root fragment. Panel (**d**) illustrates a *N. pachyderma* specimen extending a filopodia-like projection. Panels (**e**) and (**f**) show two *N. pachyderma* specimens exhibiting fully extended ectoplasmic roots. The maximum diameter of the specimens in panels (b) and (f) measured 210 and 251.5 μm, respectively. Black arrows indicate the rhizopodial activity. The contrast in the pictures has been artificially enhanced to visualize the reticulopodia and the other structures. Scale bars: 100 μm.

Photographic evidence of ectoplasmic roots (as defined in Wollenburg *et al*., 2021) was obtained for two specimens of *N. incompta* cultured at a salinity of 31. For one specimen (Fig. 1), it was possible to capture the root formation process, while for the other, the root structure could only be observed as a torn fragment (Fig. 2c). The torn root fragments and root formation were observed after 11 days in the culture. The roots, easily distinguishable from inadvertently introduced artificial fibers by their organic and motile base (as shown in Fig. 1c and d), exhibited a significant thickness and demonstrated a remarkable extension. They ranged from three times (515 μm) to more than five times (885.7 μm) the size of the specimen (as depicted in Fig. 1a and e), securely anchoring it to the walls of the flask. Interestingly, the specimen in Fig. 1 built the new root in the same position as the previously torn one (Fig. 1b). Specimen’s rhizopodia were extended before, during and after root formation. The root projection of the specimen in Fig. 1a appears to be composed of several fiber-like fragments of ectoplasm that have intertwined to form a robust structure. After a few days, this stable root structure detached from the specimen and could be observed floating next to the foraminifera in the culture flask (Fig. 1b).

### Non-permanent structures in *N. pachyderma*

**Fig. 3:**
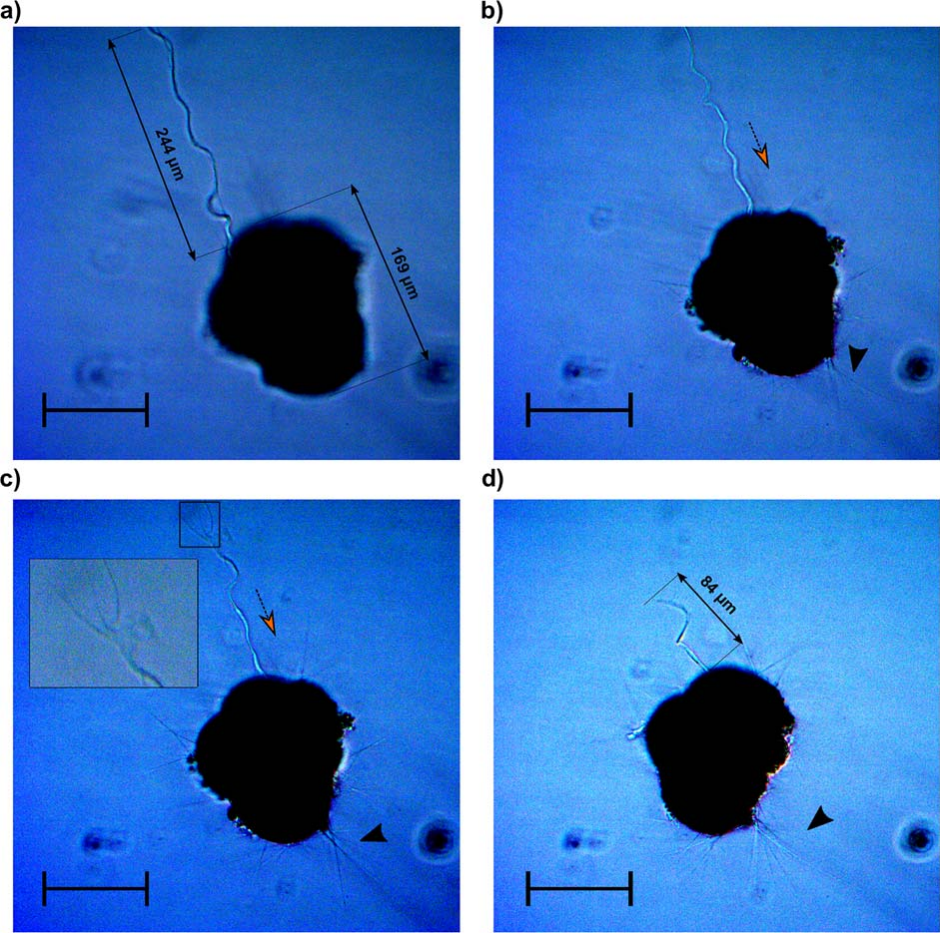
Time-lapse microscopy images of a specimen of *Neogloboquadrina pachyderma* projecting a filopodia-like structure. Panels (**a**) to (**d**) depict snapshots of a 3-min process (see video on figshare 10.6084/m9.figshare.22665031) that occurred 26 days after the foraminifera was introduced to its culture. The inset in panel (**c**) shows a close-up of the distal end of the projection. The orange arrow indicates the direction of the movement. The black arrows indicate the rhizopodial activity The contrast in the images has been artificially enhanced to visualize the filopodia-like projection. Scale bars: 100 μm.

**Fig. 4:**
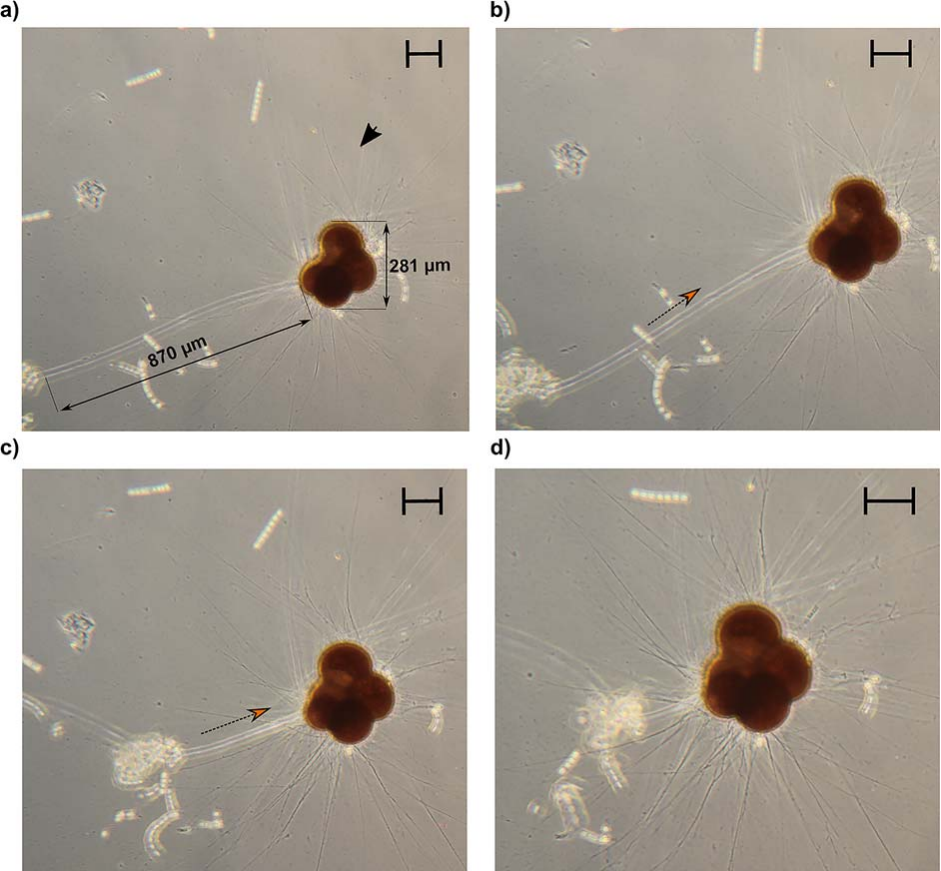
Time-lapse microscopy images of a specimen of *Neogloboquadrina pachyderma* using a filopodia-like structure to collect and gather food particles close to its position. The orange arrow indicates the direction of the movement. The black arrow indicates the rhizopodial activity. The contrast in the images has been artificially enhanced to visualize the filopodia-like projection. Scale bars: 100 μm.

Several specimens of *N. pachyderma* displayed ectoplasmic structures that differed from roots in both structure and durability. We could divide them into two categories using the term “twig-like structures”, referring to the long and thick arrangement of rhizopods (Fig. 2a and b, Supplementary Fig. 1), and the term filopodia-like structures, referring to retractile cytoplasm protrusion (Figs 2d– 4, Supplementary Fig. 1b, video on figshare: 10.6084/m9.figshare.22665031). Both types of structures were retractable as opposed to the ectoplasmic roots that were not reabsorbed once extended. Twigs-like structures could reach up to four times the size of the shell, with their distal extremity anchored to the flask wall. At their proximal end, we could observe modified rhizopods converging and eventually generating the twigs (Supplementary Fig. 1a–d). They were systematically associated with food particles at their surface.

The filopodia-like projections observed in Fig. 3 appeared to be extremely dynamic and thicker than the twigs. Furthermore, no modified rhizopods could be observed at their proximal extremity, suggesting that these structures are not associated with a specialized attachment mechanism. The specimen in Fig. 3 entirely retracted the structure in less than three minutes, accompanied by an intense lateral motion (see video on figshare: 10.6084/m9.figshare.22665031). These structures were freely moving, not attached to the jar wall at any time and were also observed in association with food particles on their surface and at their distal extremity, which appeared to be composed of at least three anastomizing filopodia (Fig. 3c). This trifurcation might have a prehensile-like function allowing the foraminifera to collect food and transport it closer to its position, as shown in the *N. pachyderma* specimen in Fig. 4.

### Ectoplasmic roots in *N. pachyderma*

Roots protrusions were also observed in three cultured specimens of *N. pachyderma* (Fig. 2d and f, Supplementary Fig. 1b). Even if no time series of the observation was recorded, the photographed structures clearly resemble the ones observed for *N. incompta*, differing in both extension and thickness from other rhizopodia. In fact, the projections measured more than two times the shell of the specimens and were found to be sticky (Fig. 2f), promoting the aggregation of particles around the foraminifera. These ectoplasmic roots were observed to be attached to the walls of the flask, providing anchorage for the specimens. In all instances, the observation of roots occurred after more than 4 days, before the water in the flask was changed. For one specimen (Supplementary Fig. 1b), the extension of the root structure changed its overall buoyancy leading to its floatation. As for *N. incompta*, the rhizopodial activity was evident when these specimens extended the structures.

### Twigs in *G. bulloides*

**Fig. 5:**
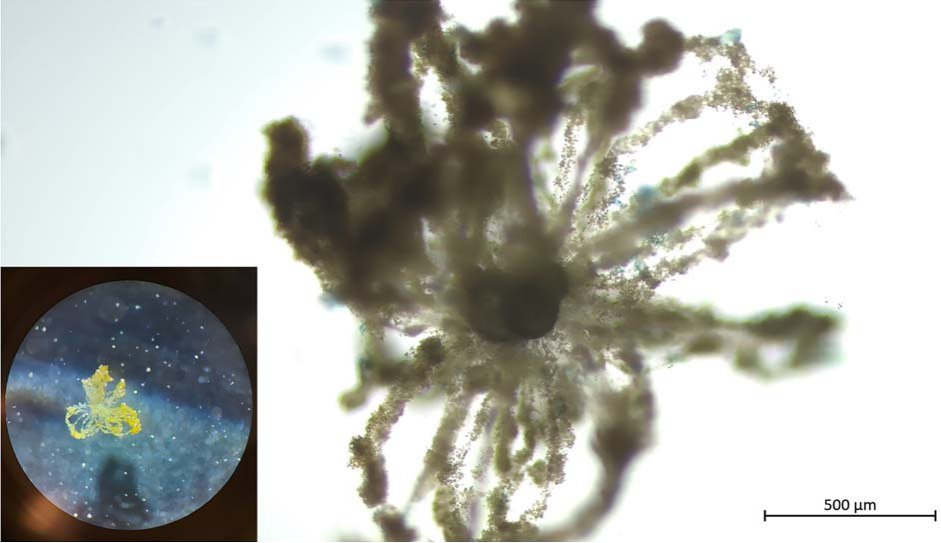
Twigs on a specimen of *Globigerina bulloides.* The inset shows a photograph of the microscope ocular, indicating that the twigs were extended, while the foraminifera was suspended in the culture flask. Scale bar: 500 μm.

Fully extended twigs consisting of anastomizing rhizopodia and algal particles were observed surrounding a specimen of *G. bulloides* after 18 days in culture (Fig. 5). These twigs stretched well beyond the spines of the specimen, likely using them as a base, and formed loop structures. Interestingly, although in the benthic genus *Cibicidoides*, these twigs were extended when the foraminifera was attached to the flask walls, *G. bulloides* presented these protrusions while suspended in the flask medium.

## DISCUSSION

### Potential origins of ectoplasmic projections

The presence of previously undocumented structures in planktonic foraminifera raises the question of whether they are genuine biological structures or merely artifacts of culture flasks. However, the fact that these structures were observed across multiple years and expeditions, and in specimens of different species and populations, provides strong evidence for their authenticity. This is further supported by the presence of similar structures in benthic lineages of the order Rotaliida (Wollenburg *et al*., 2021). Recent molecular investigations have suggested that the current diversity of planktonic foraminifera is the result of multiple invasions of the water column by benthic taxa from the order Rotaliida (Morard *et al.*, 2022). In addition, a previous experiment documented benthic-like behaviors, such as reorientation, crawling and burrowing, in the planktonic foraminifera species *Globigerinella siphonifera* (Spinose) and *Globorotalia menardii* (Non-Spinose) cultivated in the presence of sediment (Hilbrecht and Thierstein, 1996). Thus, a shared toolkit of projections between benthic and planktonic species can be expected. These structures could represent evolutionary relics deriving from planktonic foraminifera’s benthic ancestors, which could have potentially assumed novel functions in the pelagic environment. The functionality of roots and twigs observed in the benthic genus *Cibicidoides* has been previously explored in the ecological context of the investigated species, particularly with regard to their deep-sea habitat and epibenthic lifestyle. Specifically, these projections are thought to be used by the deep-sea sediment-dwelling taxa to anchor themselves in the presence of strong currents (Wollenburg *et al*., 2021). In the following sections, we will discuss the potential functions of these structures in the pelagic species *N. pachyderma*, *N. incompta* and *G. bulloides*, and their ecological implications.

### Potential functions of the observed structures

Our observations indicate that species of planktonic foraminifera belonging to the genera *Neogloboquadrina* and *Globigerina* can build ectoplasmic systems, as recently reported in the benthic taxa *Cibicidoides wuellerstorfi*, *Cibicides lobatulus* and *Cibicidoides pachyderma* (Wollenburg *et al*., 2021).

The presence of ectoplasmic roots was confirmed in two specimens of *N. incompta* and three specimens of *N. pachyderma*. As reported in benthic species, these structures are permanent once extended and are the longest and the thickest we observed. Furthermore, the sequence of photographs in Fig. 1 shows how roots can be rebuilt within 24 hours if torn, consistent with what was previously observed for benthic lineages (Wollenburg *et al*., 2021). Although we cannot resolve the molecular composition of the ectoplasmic roots from our observations, we note that they appeared in the absence or ahead of flask water exchange, leading us to believe that the amount of algal particles present affects the ability of the foraminifera to build these protrusions. From our observations, it is clear that the ectoplasmic roots help the specimen anchor to the culture flasks, providing support to its rhizopodial network that can be extended canonically or arise from the structures themselves (Fig. 2c).

The twig-like structures detected in three specimens of *N. pachyderma* are non-permanent projections that are considerably long (>700 μm). Although they are not identical to the twigs described by Wollenburg *et al*. (2021), as these specimens could retract them, we believe they serve a similar function in stabilizing the specimen while the rhizopodial network is extended and assisting in the food gathering of the reticulopodia, as shown in Fig. 2a and b, where specimens display a fully extended network of reticulopodia. Conversely, the twigs observed in *G. bulloides* closely resemble, in both extent and shape, the benthic twigs observed in *C. pachyderma*, also because of the fact that they are directed in the water column once formed (Wollenburg *et al*., 2021). Indeed, the projections are visible even at minimal magnification (Fig. 5) and are extended while the specimen is floating in the culture medium, not attached to any of the flask walls. This might suggest that by increasing the drag, twigs, similarly to calcareous spines (on which they are based), could help the foraminifera maintain its position in the water column.

To our knowledge, filopodia-like structures have previously not been photo-documented for any species within the phylum and we observed them in three specimens of *N. pachyderma*. These protrusions seem rather flexible and appear to swing during retraction causing the specimen to spin about its axis with some displacement (see video on figshare 10.6084/m9.figshare.22665031). The distal extremity magnified in Fig. 3c indicates that these projections actually consist of multiple structures merged in a slender protrusion. However, contrary to reticulopodia, filopodia-like projections do not ramify, hinting at a different cytological composition. Among the structures presented, these are the ones more directly involved in the motility of the foraminifera (see video on figshare 10.6084/m9.figshare.22665031). In absence of analogous observations within the phylum, we looked for resembling structures in other microbial eukaryotes to make inferences on the potential functions of filopodia-like projections.

Similar cytoplasmic structures have been reported in diatoms where they have been described as “cytoplasmic strands”, functioning as spatial determinants for cells and male gametes (Pollock and Pickett-Heaps, 2005; Davidovich *et al.*, 2012). Thus, it is possible that filopodia-like projections function as sensory structures that help the foraminifera search and attach to a target surface far from its position. Another possibility is that filopodia-like projections are the foraminiferal analogous to the axoflagellum described in radiolarians (Ichinohe *et al.*, 2018, 2019). The axoflagellum is long, a thick pseudopodium that radiolaria use not only for capturing food particles but also for reorienting the cell in presence of a current (Ichinohe *et al.*, 2018, 2019). In this scenario, filopodia-like protrusions may enable foraminifera to adjust their buoyancy by projecting them to realign themselves in response to changes in water flow.

### Ecological significance of our observations and outlook for future research

Most planktonic foraminifera species are omnivorous, and use their spines and/or extended reticulopodia to ensnare food particles or capture their prey (Schiebel and Hemleben, 2017). Thus, as passive drifters, the efficiency of planktonic foraminifera feeding strategy highly relies on random encounters with their food source. However, in culture, specimens of the Non-Spinose species *Globorotalia truncatulinoides* have been observed using their rhizopodial network to move from a position where they had consumed food to another one rich in diatoms (*Nitzschia* spp.) (Schiebel and Hemleben, 2017). Such behavior might indicate that planktonic foraminifera have receptor structures allowing them to detect a food source (chemosensing) and the capacity to displace toward it. This observation is consistent with filopodia-like structures functioning as sensory projections that help planktonic foraminifera reorient toward the preferred food source or collect and gather food particles (see Fig. 4).

The presence of ectoplasmic twigs as the ones we observe in *G. bulloides* could also impact the planktonic foraminifera feeding strategy. As shown in Fig. 5, twigs significantly increase the overall size of the foraminifera, multiplying, in this way, the chances of potential prey encounters. Like foraminiferal spines, pseudopodia and ectoplasmic structures in planktonic foraminifera, such as those found in *G. bulloides*, may provide a competitive advantage in low-density prey environments (Grigoratou *et al.*, 2021).

In addition, the projection of root protrusions could increase the likelihood of mate encounters in planktonic foraminifera, despite their low abundance and patchy distribution in the global oceans (Keeling and del Campo, 2017). These organisms primarily reproduce sexually by releasing hundreds of thousands of gametes. A recent investigation into their reproduction dynamics, based on mathematical modeling, has shown that the spatial concentration of gamete release is critical for the successful production of zygotes, overcoming the limitations that result from their sparse distribution (Weinkauf *et al.*, 2022). Wollenburg *et al*. (2021) demonstrated that the production of roots from multiple benthic individuals can result in the combination of these structures into a single braid-like ectoplasmic root, connecting the specimens for months and they sometimes used it to reposition themselves. As we cultured specimens individually, we can only speculate that the connection of two or multiple foraminifera specimens in braid-like roots can occur in planktonic species as well, potentially representing a significant advantage for successful reproduction events in the water column by granting spatial concentration of gamete release. Furthermore, the previously inferred potential propensity of planktonic foraminifera to descend and congregate in the chlorophyll-dense deep layer during gamete production and release may facilitate this process (Bijma and Hemleben, 1994; Schiebel *et al.*, 1997).

Planktonic foraminifera are among the most represented groups associated with sinking particles, according to a recent metagenomic investigation based on sediment trap samples (Boeuf *et al.*, 2019). We have exclusively observed ectoplasmic roots, which play a crucial role in anchoring foraminifera firmly to their substrate (Figs 1 and 2), in Non-Spinose species that are known to feed on detritus material found in marine aggregates (Greco *et al*., 2021). Based on our observations, we hypothesize that the development and utilization of roots represent a primary feeding strategy for these organisms, enabling them to extract vital nutrients from the surrounding marine snow and maintain a stable position in the turbulent ocean environment. Furthermore, the high concentrations of particles in the flask coincided with the projection of roots by the specimens, providing further support for our hypothesis. Interestingly, the high concentration of nutrients and the projection of these structures in both *G. bulloides* (twigs) and *N. pachyderma* (ectoplasmic roots) could cause a change in the buoyancy of the foraminifera.

Moreover, the ectoplasmic projections observed in planktonic foraminifera may have significant implications for the marine carbon cycle. These structures, extruded by the organism and potentially left behind as it drifts through the water column, can become incorporated into marine aggregates and enhance the vertical flux of organic matter to the deep ocean. This is especially significant as foraminifera are already recognized as major contributors to the carbon pump, particularly in the inorganic carbon pathway (Neukermans *et al.*, 2023).

It is important to note that the observations presented in this study represent the initial step toward the understanding of the functions of the described structures of planktonic foraminifera. To gain a better understanding of these structures, future investigations could employ techniques such as live actin staining (Tyszka *et al*., 2019) to elucidate the cellular structure and composition of these extensions. Additionally, the function of the described structures could be further studied by implementing different experimental designs to culture foraminifera under various conditions (e.g. different particulate concentrations) and with different substrates to uncover the factors that regulate the different types of projections. The contribution of filopodia-like projections and twigs to the buoyancy of foraminifera could be recorded and tested by placing single specimens in glass cells, similar to the experimental setup in Ichinohe *et al*. (2019). Ideally, these experiments could be combined with genomics and transcriptomics approaches to understand the genetic and molecular basis of the formation and function of the different structures.

## CONCLUSIONS

In this study, we report the presence of previously unnamed types of endoplasmic and ectoplasmic projections in three species of cold-water planktonic foraminifera: *N. pachyderma*, *N. incompta* and *G. bulloides*. Our experiments revealed two types of projections: permanent and non-permanent. Some of these structures, such as ectoplasmic roots, twigs and twig-like projections, closely resemble to those recently described in benthic taxa *Cibicides* and *Cibicidoides*, suggesting that they may play a role in anchoring planktonic foraminifera to a substrate or enhancing the chances of prey and mate encounter. Additionally, we discovered a previously undescribed, non-permanent structure that we named the filopodia-like projection in the species *N. pachyderma*. We hypothesize that this species uses this projection to locate food particles or to reorient itself in the presence of a current. The existence of roots, twigs and filopodia-like projections broadens the trophic toolkit of planktonic foraminifera. Our findings highlight the importance of life-history observations of this group and the need for further studies on these structures that could inform trait-based models (e.g. Grigoratou *et al.*, 2019), improving our understanding of the role of planktonic foraminifera in the carbon cycle.

## Supplementary Material

Supp_figure_1_fbad031

Supp_figure_1_Legend_fbad031

## Data Availability

Video in supplement to this article is available on FigShare (10.6084/m9.figshare.22665031).
