# Supplementary figures and images for: Uncovering hidden structures: previously undescribed pseudopodia and ectoplasmic structures in planktonic foraminifera
Source: J Plankton Res. 2023 Jul 5;45(4):652–60. doi: 10.1093/plankt/fbad031 (PMC10361809; doi:10.1093/plankt/fbad031)

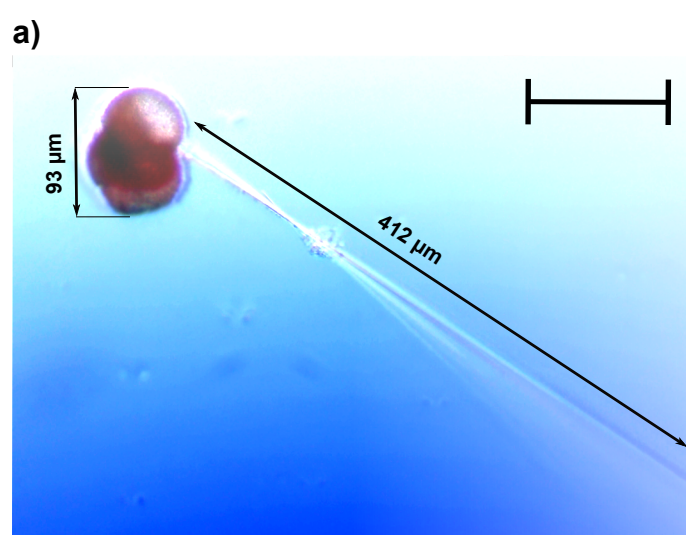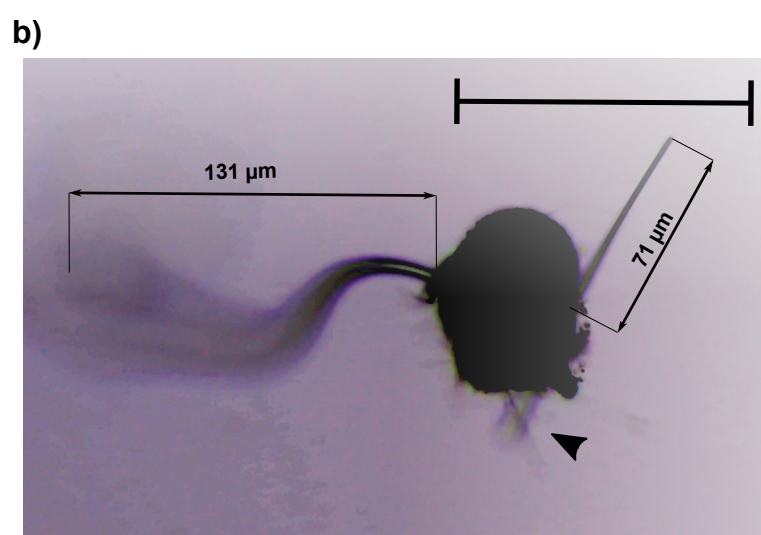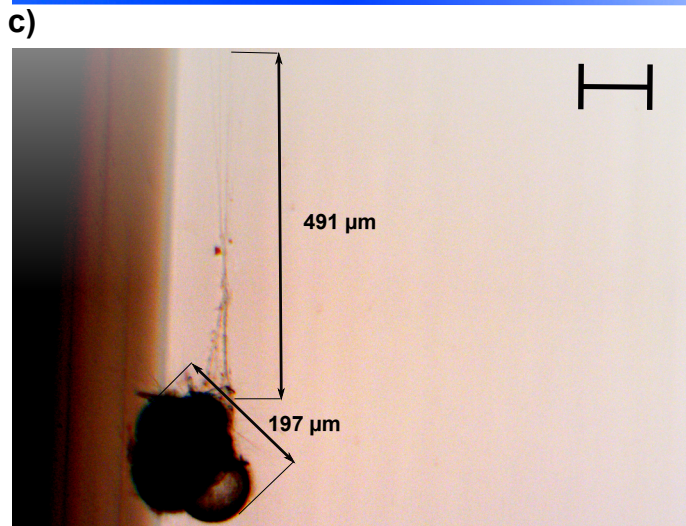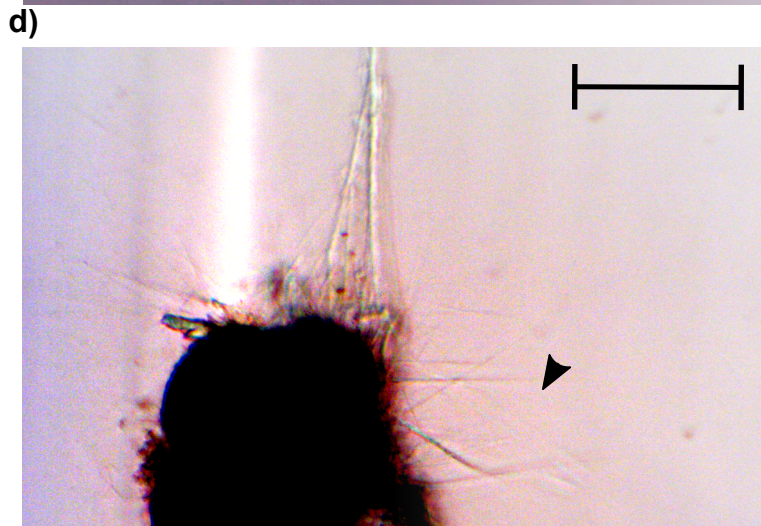

Supplement: Supp_figure_1_fbad031 [file supp_figure_1_fbad031.pdf]
