# Supplementary material for: Uncovering hidden structures: previously undescribed pseudopodia and ectoplasmic structures in planktonic foraminifera
Source: J Plankton Res. 2023 Jul 5;45(4):652–60. doi: 10.1093/plankt/fbad031 (PMC10361809; doi:10.1093/plankt/fbad031)
Supplement: Supp_figure_1_Legend_fbad031 [file supp_figure_1_legend_fbad031.docx]

**Supp. Figure 1** The observation of permanent and non-permanent structures in *N. pachyderma* is depicted in panels a), c) and d). The images show two *N. pachyderma* specimens with extended twig-like structures and projections that converge to eventually generate the twig, as shown in close-up panel d). Panel b) displays a specimen with both an ectoplasmic root and a swinging filopodia-like projection. Black arrows indicate the rhizopodial activity. Contrast in the pictures has been artificially enhanced to visualize the reticulopodia and the other structures. Scale bars: 100 μm.
